# Acute Exercise and Motor Memory Consolidation: The Role of Exercise Timing

**DOI:** 10.1155/2016/6205452

**Published:** 2016-07-03

**Authors:** Richard Thomas, Mikkel Malling Beck, Rune Rasmussen Lind, Line Korsgaard Johnsen, Svend Sparre Geertsen, Lasse Christiansen, Christian Ritz, Marc Roig, Jesper Lundbye-Jensen

**Affiliations:** ^1^Department of Nutrition, Exercise and Sports, University of Copenhagen, 2200 Copenhagen, Denmark; ^2^Department of Neuroscience & Pharmacology, University of Copenhagen, 2200 Copenhagen, Denmark; ^3^Department of Neurological Surgery, The Miami Project to Cure Paralysis, University of Miami, Miami, FL 33136, USA; ^4^Memory and Motor Rehabilitation Laboratory (MEMORY-LAB), Jewish Rehabilitation Hospital, Montreal Center for Interdisciplinary Research in Rehabilitation (CRIR), Montréal, QC, Canada H7V 1R2; ^5^School of Physical & Occupational Therapy, Faculty of Medicine, McGill University, Montreal, QC, Canada H3G 1Y5

## Abstract

High intensity aerobic exercise amplifies offline gains in procedural memory acquired during motor practice. This effect seems to be evident when exercise is placed immediately after acquisition, during the first stages of memory consolidation, but the importance of temporal proximity of the exercise bout used to stimulate improvements in procedural memory is unknown. The effects of three different temporal placements of high intensity exercise were investigated following visuomotor skill acquisition on the retention of motor memory in 48 young (24.0 ± 2.5 yrs), healthy male subjects randomly assigned to one of four groups either performing a high intensity (90% Maximal Power Output) exercise bout at 20 min (EX90), 1 h (EX90+1), 2 h (EX90+2) after acquisition or rested (CON). Retention tests were performed at 1 d (R1) and 7 d (R7). At R1 changes in performance scores after acquisition were greater for EX90 than CON (*p* < 0.001) and EX90+2 (*p* = 0.001). At R7 changes in performance scores for EX90, EX90+1, and EX90+2 were higher than CON (*p* < 0.001, *p* = 0.008, and *p* = 0.008, resp.). Changes for EX90 at R7 were greater than EX90+2 (*p* = 0.049). Exercise-induced improvements in procedural memory diminish as the temporal proximity of exercise from acquisition is increased. Timing of exercise following motor practice is important for motor memory consolidation.

## 1. Introduction

The consolidation phase of motor skill learning involves the transformation of memories from a trace or engram [1–6], established through an encoding or acquisition phase, to a more enhanced or stabile long-term memory [1, 7]. Motor memory plasticity appears to have a significant time-dependent component required for the consolidation of a trained skill or movement [8, 9] and the potential interactions between exercise and sleep may well influence the consolidation process but are currently unknown. The interplay of factors during consolidation including rest, sleep, alternative tasks, and exercise is complicated as there also appears to be different temporal aspects of consolidation itself: synaptic and systems consolidation [3, 10, 11]. The different temporal phases of motor memory consolidation are therefore complex and studies are only beginning to provide evidence of how exercise may affect these phases [12] and how the underlying cellular mechanisms are affected [13].

It can be argued that exercise prior to motor skill acquisition mainly influences acquisition itself potentially via increased awareness [14, 15] and/or a priming effect, but it can possibly affect consolidation, although this is hard to differentiate [16]. However, exercise after acquisition can only affect consolidation. An acute bout of high intensity exercise has been shown to improve motor skill learning when carried out in the period following [13, 17, 18] and preceding [19] acquisition of the skill when measured with delayed retention tests.

Exercise placed after acquisition demonstrates the greatest effect on the consolidation and the strengthening of this type of procedural memory [12, 17, 18]. Increases in peripheral (and central) availability of neurotrophic factors, energy substrates, and signaling molecules are capable of influencing memory consolidation [13]. Transient changes in corticospinal excitability (CSE) and intracortical inhibition and facilitation in the primary motor cortex, relating to motor skill learning, have also provided evidence to suggest that these mechanisms may be enhanced or protected by exercise [20–22]. Exercise, therefore, has the potential to influence memory processes by affecting the underlying cellular mechanisms and networks resulting in robust, long-term behavioural changes and thereby functioning as an endogenous neuroenhancement strategy to facilitate motor learning [23]. The literature supports the idea that exercise intensity plays a central role for improvements in motor performance [15, 24], but the importance of the temporal placement of exercise in relation to motor practice has not been thoroughly investigated.

The temporal window for interfering with the consolidation of motor memory has been investigated. Muellbacher and coworkers demonstrated that repetitive transcranial magnetic stimulation (rTMS) caused interference when placed immediately following motor learning but not 6 h later [11] and more recently Lundbye-Jensen and coworkers reported a shorter period of 3-4 h after which rTMS did not interfere with consolidation [25]. Following practice, the newly formed motor memory is susceptible to interference in a time-dependent manner from other motor tasks competing for the same neural resources and suprathreshold rTMS, both resulting in impaired performance on subsequent retention test [11, 25]. This raises the question as to whether there exists a temporal gradient relating to the effects of the exercise bout or perhaps a window of opportunity for affecting the transient plastic neural mechanisms, after which the effect disappears or is greatly reduced. The idea that this is the case has been proposed in various studies: so-called occlusion of long-term potentiation- (LTP-) like plasticity [26, 27] where there is competition for neural resources. Similarly, the synaptic tagging and capture hypothesis proposes that the initiation of LTP creates only the potential for lasting changes in neuronal structures [28, 29].

In rodents, a recent study by Siette and coworkers indicated that close temporal proximity of exercise was central to enhancing the acquisition, extinction, and reconsolidation of context conditioned fear compared to delayed exercise [30]. Memories created through fear-conditioning protocols in rodents differ, however, from memories created through motor skill practice in humans as in the present study. Nevertheless, the result is the first to highlight the importance of exercise timing. Furthermore, Xu and coworkers demonstrated in mice that a rapid (<1 h) synaptic reorganization occurs immediately following motor skill learning [31]. The results from these studies provide preliminary evidence for exercise placement in close temporal proximity to acquisition in order to have a positive effect.

In order to investigate the role of exercise timing on motor memory consolidation, we varied the temporal proximity of a high intensity exercise bout after motor skill acquisition and then measured performance changes with delayed retention tests. The primary hypothesis for this study was that a temporal gradient exists relating to the positive effects of exercise on procedural memory. This is based on the studies showing that protein synthesis and LTP, relating to memory formation, occur transiently immediately following learning [32]. Therefore, we propose that the closer the temporal proximity of the acute exercise bout to acquisition, the larger the positive effect on the retention of the procedural memory. Confirmation of this hypothesis would provide us with new important information relating to the importance of exercise timing in relation to aiding learning.

## 2. Materials and Methods

### 2.1. Study Design

**Figure 1:**
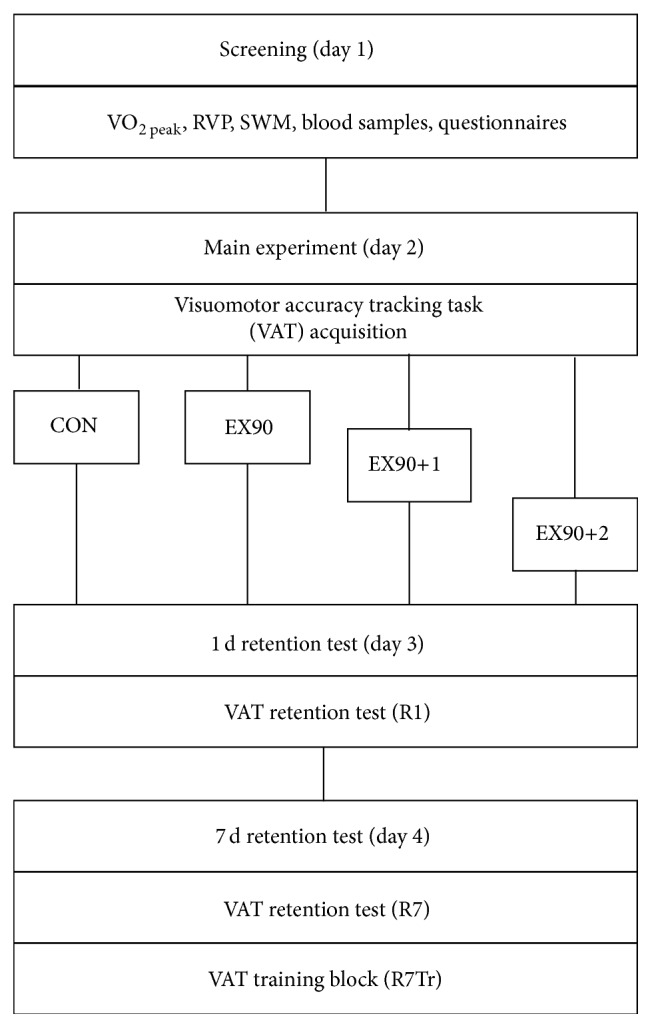
Schematic illustration of the study design. Subjects reported to the laboratory 4 times. Screening (day 1) involved baseline testing. The main experiment included acquisition of the VAT and the intervention. Retention tests were conducted at exactly 1 d (R1) and 7 d (R7) after main experiment. VO_2  peak_ = maximal oxygen consumption; RVP = rapid visual processing (sustained attention). SWM = spatial working memory.

A schematic illustration of the study design is shown in Figure 1. Subjects were required to visit the laboratory on four separate occasions with the aim of assessing the effects of a high intensity exercise bout on the consolidation and retention of a newly acquired motor skill. The first visit involved screening for preliminary and baseline measurements. Subjects performed a graded maximal exercise test on a bicycle ergometer to measure their maximal oxygen uptake (VO_2  peak_).

At least 1 d after the screening session, subjects returned to the laboratory to complete the main experimental session and were required to refrain from exercising during this period. Subjects then returned to the laboratory exactly 1 d and 7 d after the main experiment to complete the retention tests, R1 and R7, respectively. All sessions were carried out at the same time of day (±2 h). Randomization was stratified to ensure that the groups were matched for age, body mass index (BMI), relative maximal oxygen consumption (VO_2  peak_: mL O_2_·min^−1^·kg^−1^), and baseline score in the visuomotor accuracy tracking task (VAT). The subjects were divided between a resting control group (CON) and three exercise groups (EX90, EX90+1, and EX90+2). Data from subjects in the CON and EX90 have been reported in a previous study focusing on the importance of exercise intensity [18]. The subjects were required to abstain from physical activity 2 h before and 4 h after the test sessions [17]. They were also required to refrain from caffeinated products in the same time frame [33].

### 2.2. Subjects

Forty-eight able-bodied, healthy, right-handed males (24.0 ± 2.5 yrs) were recruited from the Copenhagen area to participate in the study (Table 1). Right-handedness for each subject was evaluated with the Edinburgh Handedness Inventory (87.4 ± 2.7) [34]. At the time of recruitment for the study all subjects were naïve to the VAT used to investigate motor skill learning and procedural memory. Exclusion criteria for participation in the study included the following: age below 20 or over 35, body mass index (BMI) above 30, a history of neurological, psychiatric, or medical diseases, and a current intake of medication and/or recreational drugs, which could have an impact on learning and/or the central nervous system. All subjects gave their written informed consent prior to testing. The experiments were approved by the local ethics committee for the Greater Copenhagen area (protocol H-2-2011-032) and the study was performed in accordance with the declaration of Helsinki.

### 2.3. Graded Maximal Exercise Test

The graded maximal exercise test was conducted in order to assess the subject's aerobic fitness level and to collect blood lactate samples at various workloads. The test was conducted following the protocol used by Roig et al. [17]. Maximal oxygen consumption was determined when at least one of the following criteria was met: a plateau in the VO_2_ curve, a RER ≥ 1.1, an inability to maintain 80 RPM, and/or volitional exhaustion. Mean values for relative VO_2  peak_ and *W*_max_ for each group can be seen in Table 1.

All subjects performed standardized neuropsychological tests of spatial working memory (SWM) and sustained attention (rapid visual processing, RVP) with CANTAB software (Cambridge Cognition Ltd., UK). Questionnaires were completed including the Academic Motivation Scale (AMS) [35], the Task and Ego Orientation in Sport Questionnaire (TEOSQ) [36], the International Physical Activity Questionnaire- (IPAQ-) Long [37], flow proneness [38, 39], health background via a standardized general eligibility questionnaire, and positive and negative affective status (PANAS). No differences were observed between groups for RVP (sustained attention), SWM (Table 2), and IPAQ levels at baseline (Table 1).

### 2.4. Main Experiment and Retention Tests

On arrival at the laboratory subjects were required to complete the PANAS [40] to determine positive (PAS) and negative (NAS) affects before VAT acquisition, R1, and R7, respectively. Subjects also completed the Stanford Sleepiness questionnaire [41] prior to starting motor skill learning. The VAT [17] involved 5 training blocks (B1, B2, B3, B4, and B5) of 20 identical targets (4 min per block) with feedback in the form of a performance score, separated by 2 min rest periods between blocks. Following the completion of block 3 subjects completed a flow questionnaire relating to how they evaluated their mental state and performance during the VAT [42]. This was the Danish version of the 13-item Flow Kurz Skala [43], which has been previously used and described by this group [39]. Similarly an Intrinsic Motivation Inventory (IMI) [44] was filled out on completion of the VAT.

At 20 min after VAT the subjects assigned to the control group (CON) remained seated. Subjects in group EX90 completed a standardized acute exercise bout on a cycle ergometer in an adjacent laboratory. At 1 h and 2 h after VAT subjects in groups EX90+1 and EX90+2 completed the same exercise bout, respectively. In the period following completion of the VAT and start of exercise, subjects sat quietly in an adjacent office and were allowed to read nonacademic literature (newspapers and magazines were provided). The exercise bout consisted of a short warm-up followed by 3 × 3 min intervals at 90% of peak power output (*W*_max_) for all exercise groups. Blood lactate measurements were taken before and 5 min after exercise as well as in the last 30 s of each of the three intervals. The main experiment was concluded with completion of the Montréal Sleep Diary for the night preceding the main experiment. This was an adapted sleep questionnaire based on the Pittsburgh Sleep Diary [45], which has been used in numerous studies [46–48].

At 1 day after acquisition subjects were required to complete a retention test in the VAT (R1), without feedback on motor performance. The removal of feedback at the retention tests was done to exclude any learning effects, which might relate to receiving feedback such as guidance and motivation [49]. This format was also repeated at the 7 d retention test (R7) and an additional training block (R7Tr) in the VAT with feedback was completed in order to check for continued learning potential. Both retention tests were concluded with the Montréal Sleep Diary.

### 2.5. Exercise Protocol

The exercise protocol was identical to the one used in the study by Thomas and coworkers [18]. The protocol was designed to ensure high levels of peak blood lactate (≥10 mmol/L [17, 50]) and the total duration of the exercise was limited (17 min) in order to avoid excessive fatigue and/or dehydration, which could potentially have a negative effect on memory processing [51, 52]. Subjects performed a two-minute warm-up on a cycle ergometer (Ergomedic 939E, Monark, Sweden) at 100 W followed by a further 2 min at 60% of *W*_max_ after which they completed 3 blocks of high intensity cycling (90% of *W*_max_) keeping a cadence of ≥80 RPM interspersed by a 2 min lower intensity active rest interval (60% of *W*_max_) also with a cadence of ≥80 RPM.

The exercise session lasted exactly 17 min including the warm-up. Heart rate, via heart rate monitors (Polar Electro, Kempele, Finland), and rating of perceived exertion (RPE) values (Borg Scale) [53] were recorded during exercise and blood lactate measurements (Accutrend® Plus System, Roche Diagnostics, Switzerland) were taken at rest prior to exercise (pre), at completion of each work interval (1, 2, and 3) then again at 5 min after exercise completion (POST).

### 2.6. Visuomotor Accuracy Tracking Task (VAT)

**Figure 2:**
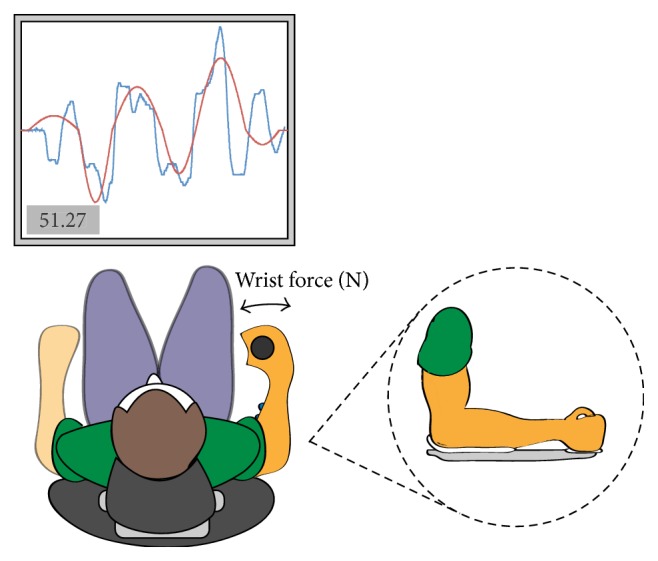
Illustration of the setup for the visuomotor accuracy tracking task (VAT). Subjects were seated at a table in front of a computer screen with their right forearm secured in a custom made setup. A red target trace appeared on the screen and a blue trace cursor moved from left to right with a constant velocity. The handle translated medial and lateral torque force into a deflection of the cursor trace either up or down. Subjects were instructed to follow the target trace as closely as possible. A motor performance score was presented at the completion of each trial during motor practice.

The VAT has been described in detail previously by this group [13, 17] and the protocol used was identical [18]. All subjects performed a standardized familiarization. An illustration of the VAT setup can be seen in Figure 2. Each VAT trial consisted of a fixed target consisting of a modified triple sine wave curve. Subjects were required to track the target as accurately as possible by moving a cursor trace up and down, respectively, with wrist extension moving the cursor upwards and flexion moving it downwards. At the end of each trial, augmented feedback on performance was presented as a numerical motor performance score and the subject's trace presented with the target trace. The numerical score range was 0–100 with 100 representing a perfect trace of the target. Augmented feedback was only presented during motor skill acquisition, not during delayed retention tests [49]. Trials were separated by a 1 s pause.

Subjects performed the VAT on four occasions: at the main experiment (acquisition), at the 1 d retention test (R1), at the 7 d retention test (R7), and an additional motor skill training block at 7 d (R7Tr), which was included to check for a ceiling effect in the VAT. The acquisition phase consisted of 5 blocks of 20 trials (100 trials in total) with each block taking 4 min to complete, with rest periods of 2 min between blocks giving a total time of 28 min. Performance scores for trials 2–20 in B1–5 were used for statistical analysis. The two retention tests (R1 and R7) consisted of 1 block of 20 targets with mean scores for targets 2–20 used for analysis. The test at 7 d to check for a ceiling effect (R7Tr) was 1 block of 20 targets under the same conditions as acquisition with the mean score for targets 2–20 used for analysis. Subjects therefore performed a total of 160 trials throughout the whole experiment.

### 2.7. Statistical Analysis

Two-way repeated measurements ANOVA models were fitted by means of linear mixed models. For the VAT parameters linear mixed models including group-time interactions as fixed effects and subject-specific random effects were fitted to account for the 4-by-7 two-way analysis of variance (ANOVA) layout of the study (4 groups and 7 time points) with repeated measurements per subjects. The random effects captured inherent variability between subjects and thus allowed removal of between-subject variation. These included separate analyses for acquisition (4-by-5) and retention (4-by-3) as two-way ANOVA layouts. Within these models we identified a limited number of comparisons corresponding to the research questions of interest in this study. Model checking was based on residual plots and normal probability plots using the raw residuals. The same linear mixed model was also fitted to the exercise parameters for the three groups EX90, EX90+1, and EX90+2 with a 3-by-4 two-way ANOVA layout. Data from tests of sustained attention, spatial working memory, PANAS, sleepiness, physical activity level, and sleep were also analyzed using similar linear mixed models (also with an 4-by-3 two-way ANOVA layout).

Separate models were fitted for the VAT acquisition phase and then subsequently the retention tests R1 and R7. Within the two-way ANOVA layout of each model we identified a limited number of relevant comparisons between changes from baseline within intervention groups in order to reduce the problem of multiple testing. Additionally, we also considered comparisons between groups at specific time points. All these pairwise comparisons were evaluated using model-based *t*-tests derived from the mixed models. The resulting *p* values for comparisons of changes were multiplicity adjusted using the computational single-step method, which provides a less conservative adjustment than the Bonferroni adjustment through utilization of correlations between means entering in the pairwise comparisons [54]. The *p* values for the additional comparisons between groups at specific time points were left unadjusted. Likewise, model-based *t*-tests were used to evaluate differences in changes between group mean scores at the time points block 5 (B5), R1, and R7. Data are reported as mean ± SE unless otherwise stated, where appropriate data are reported with 95% C.I. A significance level of 0.05 was applied.

All statistical analyses were carried out in R (R Core Team, 2015). In particular, linear mixed models were fitted using the functionality of the package* lme4 *[55], whereas multiple comparisons and adjusted *p* values were calculated using the single-step method provided by the package* multcomp* [54].

## 3. Results

### 3.1. VAT Acquisition

**Figure 3:**
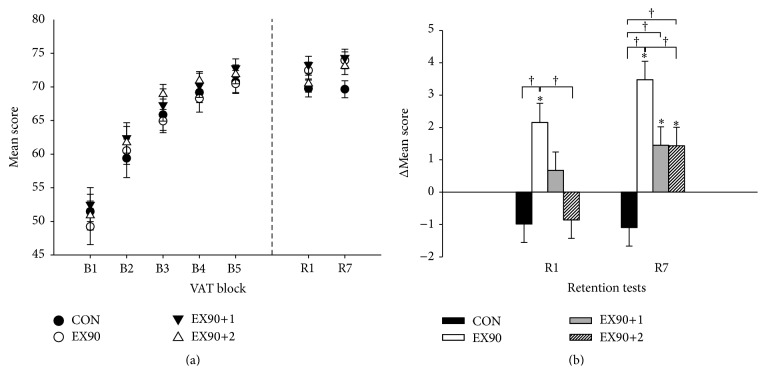
(a) Mean scores (±SE) in the VAT at acquisition blocks 1–5 and at 1 d (R1) and 7 d (R7) retention. (b) Changes in mean scores (±SE) for all groups in the VAT from B5 to R1 and R7.  ^*∗*^Significant change from B5 (*p* < 0.05).  ^†^Significant between-group difference (*p* < 0.05).

Mean scores (±SE) for all groups at all time points can be seen in Figure 3(a). From B1 to B5 all groups showed significant improvement (all *p* < 0.05), with a mean increase of 39.4 ± 1.2% equivalent to an increase of 20.6 ± 0.5 in mean performance score. There were no between-group differences in performance score changes from B1 to B5, indicating that skill improvements during acquisition and, more importantly, skill level at the end of acquisition (B5), were similar among groups (Figure 3(a)).

### 3.2. VAT Retention Tests

Changes in mean performance score from B5 to R1 for EX90 were greater than CON 3.14 ± 0.82 (*p* < 0.001) and this change for EX90 was also greater than the EX90+2 group 3.01 ± 0.82 (*p* = 0.001). From B5 to R7 there was a significant difference of 4.56 ± 0.81 (*p* < 0.001) between the EX90 and CON groups as well as between the EX90+1 and CON groups 2.54 ± 0.81 (*p* = 0.008), between the CON and EX90+2 groups 2.52 ± 0.81 (*p* = 0.008), and between the EX90+2 and EX90 groups 2.04 ± 0.81 (*p* = 0.049). There was a tendency towards a greater change between the EX90 group and the EX90+1 group between B5 and R7 2.03 ± 0.81 (*p* = 0.052).  Figure 3(b) shows the changes in mean VAT scores at R1 and R7 relative to B5.

For the EX90 group there was a significant increase of 2.15 ± 0.59 (*p* < 0.001) in mean performance score at R1 compared to B5 and likewise from B5 to R7 3.47 ± 0.57 (*p* < 0.001) (B5 70.48 ± 1.26; R1 72.63 ± 1.26: R7 73.95 ± 1.26). There was a significant increase of 1.45 ± 0.57 (*p* = 0.011) in mean performance score for EX90+1 from B5 to R7 (B5 72.79 ± 1.26: R7 74.24 ± 1.26) and for EX90+2 1.43 ± 0.57 (*p* = 0.012). At R1 a significant between-group difference of 3.69 ± 1.79 (*p* = 0.038) was observed between CON and EX90+1 (CON 69.77 ± 1.26; EX90+1 73.46 ± 1.26). At R7 significant between-group differences in mean scores of 4.29 ± 1.78 (*p* = 0.016) and 4.58 ± 1.78 (*p* = 0.010) were found between CON and EX90 and CON and EX90+1 (CON 69.66 ± 1.26; EX90 73.30 ± 1.26; EX90+1 74.24 ± 1.26), respectively, and between CON and EX90+2 3.64 ± 1.78 (*p* = 0.041).

### 3.3. Comparison of 7 d Retention R7 and Motor Performance with Continued Practice R7Tr

Changes in performance score from R7 to R7Tr for the CON group were greater than the EX90 group 2.11 ± 0.73 (*p* = 0.017) and likewise the change for the EX90+2 group was greater than the EX90 group 1.96 ± 0.73 (*p* = 0.030). There was a significant increase of 2.32 ± 0.51 (*p* < 0.001) for the CON group between time points R7 and R7Tr (R7 69.66 ± 1.22; R7Tr 71.98 ± 1.22). Likewise, for the EX90+1 group 1.94 ± 0.51 (*p* < 0.001) (R7 74.24 ± 1.22; R7Tr 76.18 ± 1.22) and the EX90+2 group 2.18 ± 0.51 (*p* < 0.001) (R7 73.30 ± 1.22; R7Tr 75.48 ± 1.22) demonstrating that learning was not saturated and that there was potential for further learning. However, this was not the case for EX90 (R7 73.95 ± 1.22; R7Tr 74.17 ± 1.22).

### 3.4. Physiological Response

**Figure 4:**
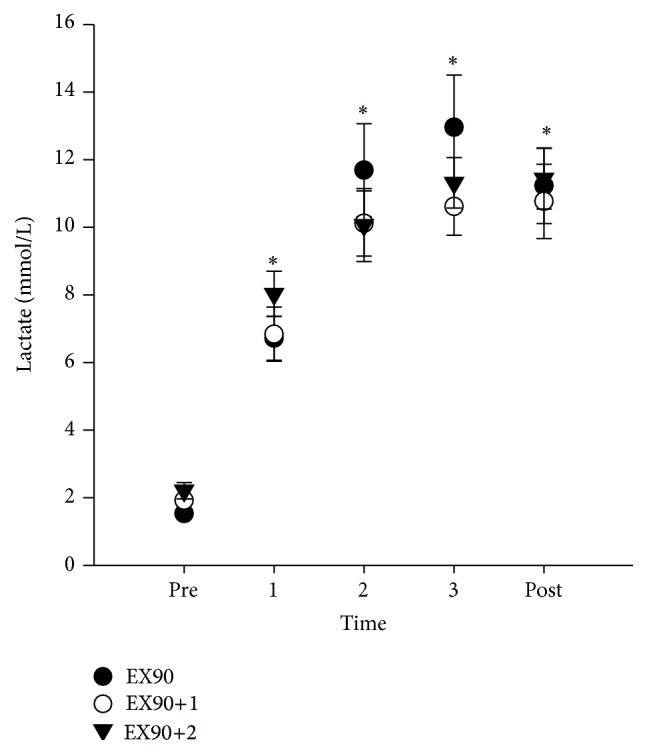
Mean blood lactate levels (mmol/L) for the three high intensity exercise groups (±SE) at time points before exercise (pre), intervals 1, 2, and 3, and 5 min after exercise (post).  ^*∗*^Significant difference compared to pre values (*p* < 0.05).

Group mean values for the exercise bouts are shown in Table 3. No significant differences between groups were observed for any exercise parameter. Blood lactate levels for time points before exercise (pre), intervals 1, 2, and 3, and 5 min after exercise (post) are presented in Figure 4.

## 4. Discussion

The main finding of this study is that a temporal gradient exists in the positive effect of an acute high intensity exercise bout on the retention of a motor skill. Exercise carried out 20 min after motor skill acquisition led to superior retention (changes in performance scores) compared to a resting control group and the delayed (+2 h) exercise group at both 1 and 7 d. Delaying exercise by 2 h appears to significantly diminish this effect, although changes in performance scores at 7 d were still greater than a resting control group. All groups were capable of improving performance in R7Tr, suggesting that a potential ceiling effect for the VAT did not exist for any group. These findings may further our understanding of how exercise plays a role as a potent amplifier of practice-dependent plasticity and demonstrates how we may be able to advance practical guidelines in rehabilitation, sports, ergonomics, and education settings in order to aid learning processes.

The current literature supports the idea of a memory trace or motor engram [2], which is established through motor skill practice and is then susceptible to positive interference, such as exercise [13, 17–19] and sleep [56]. Negative interference can also occur via tasks that compete for the same neural resources or networks [7, 25, 57, 58] or rTMS [11, 59, 60] in the period following acquisition. The present findings are in accordance with recent studies [13, 17, 19] and support the existence of a temporal window in which motor memory consolidation can be influenced. A recent finding reported by Rhee and coworkers [61] included a delayed exercise condition (~1.75 h) following a motor sequence task. This behavioural intervention resulted in a broad enhancement and protection of the offline gains when performed immediately prior to a second interfering task at 2 h. The performance level at 24 h was comparable to a control group with no interference and greater than an exercise condition immediately following the initial task. Whereas exercise immediately after practicing a visuomotor precision task is beneficial for motor memory consolidation, mechanisms underlying interference effects in sequence learning may be somewhat different. The findings by Rhee et al. do however support the notion of beneficial effects of exercise following learning. Additionally, the current findings demonstrate that enhancing memory consolidation through intense aerobic exercise may be restricted to a narrower timeframe compared to that of negative interference.

Motor skill learning is accompanied by a transient increase in corticospinal excitability (CSE) observed as increased MEP amplitude [10, 25, 62, 63]. Early work suggested a causal link between changes in CSE and the retention of motor memory [11]. More recently it has been suggested that the increase in CSE relative to the potential for increases in CSE, the so-called occlusion index, rather than the change itself relating to offline changes in motor memory [26, 64, 65]. This represents a limited ability for further LTP-like plasticity due to competition of neural resources [26] and it is argued that the greater the extent of occlusion the more resilient the memory trace to retrograde interference. These results correspond to findings by Tunovic and colleagues who observed that when CSE decreases were abolished via theta burst TMS following an implicit version of the serial reaction time task skill improvements were observed and these improvements corresponded to the changes in CSE [22]. Since exercise increases CSE [22] and affects intracortical networks [20, 21] this could be one marker for the underlying processes leading to offline changes in motor performance.

The time-dependent processes involved in establishing motor memory are critical in relation to this study's results. The synaptic tagging and capture hypothesis [28, 29] propose that the cellular component of consolidation involves a temporary structural state of the synapse, which represents a permissive unlocking process without which protein synthesis and the supply of plasticity related proteins (PRPs) are incapable of stabilizing plasticity [28]. Exercise-induced transient increases in circulating levels of brain derived neurotrophic factor (BDNF), norepinephrine, epinephrine, dopamine, and lactate [13, 66, 67] might support or even amplify the tagging process, which in turn could potentially stimulate and signal a variety of pathways supporting long-term potentiation (LTP) and an upregulation of protein synthesis. An important distinction, however, is the placement of the exercise bout. Many studies have reported effects on cognitive function when the exercise bout is placed before or during the task [67], but here we focus on motor skill learning with exercise placed after acquisition to investigate motor memory consolidation.

The transient and high (>10 mmol/L) increase in blood lactate level, which in turn signals an upregulation of the monoamine carboxylate transporters [68], a release of BDNF [13], and increases in CSE excitability [69], may represent an amplification effect that could explain the increase in the postacquisition susceptibility of the neural networks to be affected by the exercise bout. In combination with the transient increases in biomarkers such as dopamine, norepinephrine, epinephrine, and insulin-like growth factor 1 (IGF-1) [13] there may be an interaction producing a favorable cellular and molecular environment for consolidation. The catecholamine hypothesis relating to the effects of acute exercise on cognition may also provide a neurochemical basis for the improvements in procedural memory [66]. However, linking distinct brain functions across different experimental protocols in light of this study's results should be done with caution until further evidence supports this.

If the plastic mechanisms triggered by motor learning itself are transient and, as several theories suggest, are susceptible in a time frame of ~2 h after learning then it follows that the effects of exercise on consolidation would be the greatest when adding to these already upregulated processes at <1 h after motor learning. It is, however, important to underline that these lines of argumentation are speculative and at best help explain our results on the basis of current knowledge.

Offline gains in motor memory have been demonstrated [57] and investigated in order to try and understand the neurobiology of consolidation [1, 3, 4, 70–73]. Studies differentiate between different temporal phases of consolidation such as synaptic and systems consolidation [3]. Different stages, fast and slow [74], are also proposed as well as parallel mechanisms including striatal and hippocampal systems [75]: corticostriatal and corticocerebellar systems [76, 77]. There are, furthermore, different aspects of the learning task: goal versus movement [78]. Whether exercise preferentially affects one or more of these mechanisms, or all at once via a global flooding of the signaling cascades involved, can only be speculated on at the current time. Similarly, the role of sleep and the interaction between sleep and exercise is also currently poorly understood although sleep-dependent motor memory plasticity has been reported in the literature [8, 9, 14, 56]. The registration of sleep and affect schedules (PANAS) in this study showed that there were similar levels between groups and across time points effectively excluding these parameters as confounders.

### 4.1. Limitations

The study design implied an element of repeated measurements per subject such that each subject served as his or her own control, which allowed us to eliminate some of the between-subject variation and thus also increase power. Nevertheless, the relatively small sample size should be considered when interpreting the results. Furthermore, any extrapolation of these results to other types of memory and learning/exercise paradigms must be done with caution.

## 5. Conclusions

The results of this study show that the positive effects of an acute bout of high intensity exercise on the consolidation and retention of motor skill learning are greatest when placed in close temporal proximity following the acquisition of the skill with this positive effect diminishing as time to exercise increases. This temporal gradient would support the idea that in order to maximize the effects of exercise on motor memory consolidation, it should be placed immediately following acquisition of the skill/encoding of the memory (<1 h). The specific neurophysiological mechanisms, which are affected by exercise in this period, are currently not fully understood, but the systemic effect of an acute exercise bout may well amplify processes of neuroplasticity at a systemic, molecular, and cellular level.

## Figures and Tables

**Table 1:** Baseline characteristics of study subjects (mean ± SD).

	CON	EX90	EX90+1	EX90+2
Number of subjects	12	12	12	12
Age (years)	24.2 ± 3.0	24.3 ± 2.3	24.1 ± 2.3	23.6 ± 2.0
Weight (kg)	81.7 ± 10.0	77.9 ± 12.5	80.4 ± 6.7	78.8 ± 13.1
Height (cm)	185.8 ± 6.0	180.1 ± 9.1	184.0 ± 7.6	182.3 ± 7.0
BMI (kg/m^2^)	23.7 ± 2.6	23.9 ± 2.4	23.8 ± 1.9	23.6 ± 2.8
General physical activity ^#^(IPAQ) (low/moderate/high)	0/4/8	0/2/10	0/6/6	0/1/11
VO_2 peak_ (mL O_2_·min^−1^·kg^−1^)	51.0 ± 4.6	51.1 ± 4.6	49.0 ± 5.6	50.4 ± 6.9
*W*_max_ (W)	325.0 ± 50.0	320.8 ± 39.7	304.2 ± 25.8	312.5 ± 37.7
Baseline VAT score	51.5 ± 8.9	49.2 ± 9.3	52.2 ± 9.3	50.6 ± 7.8

^#^Number of subjects per group.

BMI = body mass index, IPAQ = international physical activity questionnaire (long), VO_2 peak_ = maximal relative oxygen uptake, *W*_max_ = peak power output, and VAT = visuomotor accuracy tracking task.

**Table 2:** Results for tests of sustained attention, spatial working memory, PANAS, sleepiness, physical activity level, and sleep (mean ± SE). ^*∗*^Significantly different from main experiment (*p* < 0.05).

	CON	EX90	EX90+1	EX90+2
Sustained attention (total hits)	21.0 ± 1.0	23.1 ± 1.1	22.7 ± 0.8	20.6 ± 1.5
Spatial working memory (total errors)	11.1 ± 3.6	11.9 ± 2.4	7.7 ± 2.7	11.8 ± 3.0
PANAS				
PAS				
(i) Main experiment	30.3 ± 2.2	28.7 ± 1.8	26.6 ± 1.9	29.8 ± 2.0
(ii) R1	28.9 ± 2.3	28.9 ± 2.2	30.0 ± 2.5	32.2 ± 1.7
(iii) R7	28.2 ± 2.8	28.6 ± 2.6	28.2 ± 2.1	31.0 ± 1.7
NAS				
(i) Main experiment	11.8 ± 0.6	12.3 ± 0.5	12.8 ± 1.3	11.6 ± 0.5
(ii) R1	11.7 ± 0.6	11.8 ± 0.5	11.8 ± 1.7	10.4 ± 0.2
(iii) R7	11.9 ± 1.0	10.8 ± 0.3	11.1 ± 0.7	10.0 ± 0.0
Sleepiness (main experiment)	2.6 ± 0.2	2.8 ± 0.3	3.2 ± 0.4	3.1 ± 0.3
Sleep diary (hours slept prior to)				
(i) Main experiment	7.8 ± 0.3	7.3 ± 0.4	7.4 ± 0.2	6.7 ± 0.4
(ii) R1	7.6 ± 0.3	7.6 ± 0.3	7.6 ± 0.3	7.6 ± 0.3^*∗*^
(iii) R7	7.6 ± 0.2	7.4 ± 0.3	7.4 ± 0.3	7.3 ± 0.3

PANAS = positive and negative affect schedule, PAS = positive affect schedule, and NAS = negative affect schedule.

**Table 3:** Exercise data for EX90, EX90+1, and EX90+2 groups (mean ± SE).

	EX90	EX90+1	EX90+2
Watt (W) 90% *W*_max_	285.0 ± 11.5	263.0 ± 7.5	273.8 ± 10.3
Watt (W) 60% *W*_max_	190.0 ± 7.7	175.0 ± 5.0	182.5 ± 6.9
Baseline lactate (mmol/L)	1.5 ± 0.1	1.9 ± 0.1	2.2 ± 0.2
Peak lactate (mmol/L)	13.0 ± 1.6	10.8 ± 1.1	11.4 ± 0.9
RPE (work)	17.0 ± 0.3	16.7 ± 0.3	16.6 ± 0.3
RPE (active rest)	13.7 ± 0.4	13.4 ± 0.4	13.9 ± 0.3
Work heart rate (beats/min)	173.6 ± 3.8	176.9 ± 3.2	175.4 ± 2.0
Active rest heart rate (beats/min)	152.1 ± 3.9	154.6 ± 4.1	154.8 ± 2.1

RPE = rating of perceived exertion.
